# Stressed out or subjective acquisition of competence – how do veterinary students see their curative work placement?

**DOI:** 10.3205/zma001008

**Published:** 2016-02-15

**Authors:** Marc Dilly, Andrea Tipold, Katja Geuenich

**Affiliations:** 1 University of Veterinary Medicine Hannover, Foundation, Clinical Skills Lab, Hannover, Germany; 2 University of Veterinary Medicine Hannover, Foundation, Small Animal Clinical, Hannover, Germany; 3 Park Clinic Röhrer, Academy for Psychosomatics in the World of Work, Eschweiler, Germany

**Keywords:** Veterinary studies, work placement, stress, resources, competencies, burnout, students

## Abstract

Veterinary studies in Germany are regulated by the Veterinary Certification Act (TAppV). The practical part of the education consists of 1,170 hours, whereby up to 850 hours can be spent on the curative work placement. A curative work placement can result in physical and psychological stress in the sense of a professional overload. It is the aim of this study to find out in what areas and to what extent competence is acquired and psychological stress exists in students during their work placement. Veterinary students (n=142) from all German education institutes participated in a voluntary online-study based on Burnout Screening Scales (BOSS) as well as a questionnaire regarding the acquisition of competence and excessive stress during the work placement (FKÜP). The distribution of values for work placement related stress show that such work placement related stress is generally slightly increased (T=60) and lies above that of occupational stresses within the normal population. Work placement related physical complaints also show a significant slight increase (T=61). A value (T=42) within the normal range was determined for the resource values. Few of the students questioned considered themselves to be excessively stressed in favour of a high subjective acquisition of competences. The largest increase regarding the acquisition of competence was noted for the areas of animal handling/restraint and application and injection techniques. In the sense of a perceived excessive demand regarding practical capabilities the areas of emergency management, surgery and medication dispensation were mentioned. With regard to the load structure and the acquisition of competence by veterinary students during their work placement, more support of the individual and a balancing of teaching/learning goals would be desirable and represents a promising approach.

## Zusammenfassung

In Deutschland ist das Tiermedizinstudium durch die Tierärztliche Approbationsverordnung (TAppV) geregelt. Der praktische Anteil der Ausbildung beträgt 1.170 Stunden, wobei bis zu 850 Stunden in kurativen Praktika absolviert werden. In kurativen Praktika kann es zu physischen und psychischen Belastungen im Sinne einer Arbeitsüberforderung kommen. Ziel dieser Studie soll es sein herauszufinden, in welchen Bereichen und in welchem Umfang Kompetenzen erworben und psychische Belastungen bei Studierenden im Praktikum vorhanden sind. An einer freiwilligen Online-Umfrage, basierend auf Burnout-Screening-Skalen (BOSS) sowie einem Fragebogen zum Kompetenzerwerb und Überforderungen im Praktikum (FKÜP), nahmen Tiermedizinstudierende (n=142) aller deutschen Bildungsstätten teil. Die Verteilung der Werte für praktikumsbezogene Belastungen zeigen, dass praktikumsbezogene Belastungen im Mittel signifikant leicht erhöht (T=60) sind und oberhalb der beruflichen Belastungen der Normalpopulation liegen. Ebenfalls sind die praktikumsbezogenen Beschwerden auf körperlichen Ebene signifikant leicht erhöht (T=61). Für die Ressourcenwerte wurde ein Wert (T=42) innerhalb des Normalbereiches ermittelt. Dabei schildern die befragten Studierenden wenige Überforderungsgefühle zu Gunsten eines hohen subjektiven Kompetenzerwerbs. Für die Bereiche Umgang mit Tieren, Zwangsmaßnahmen sowie Applikations- und Injektionstechniken konnten die größten Zuwächse beim Kompetenzerwerb ermittelt werden. Im Sinne eines Überforderungsgefühls bei praktischen Fertigkeiten wurden die Bereiche Notfallmanagement, Chirurgie und Medikamentenabgabe genannt. Bezogen auf die Belastungsstruktur und den Kompetenzerwerb von Studierenden der Tiermedizin im Praktikum ist mehr Förderung der einzelnen Person und ein Abgleich von Lehr-/Lernzielen wünschenswert und ein vielversprechender Ansatz.

## Introduction

Stress and psychological loads exist in all the healing professions and especially within the medical sector, and leads to substantial physical and psychological stress [1]. [2]. Burnout in the sense of a prolonged professional overload is characterised by a reduction in professional performance of physicians within the sector of human medicine, accompanied by an increased probability of committing professional errors [3]. Veterinary surgeons have also been found to suffer under substantial professional loads right down to substance abuse [4], [5], [6]. Similar problems have been found by a study carried out into university information system for the area of medical students [7]. The causes and details of changes within the private and professional environment are complex and multi-layered, especially after studies have been completed and a professional career has commenced [8], [9]. Studies carried out in the Netherlands in particular highlight the tension area of professional expectations/stresses and individual resilience of graduates [10], [11]. Further studies from Finland [12], New Zealand [8], Germany [4], [5], [13], Belgium [14], England [15], [16] and Australia [17], [18], [19] deal with the mental well-being of veterinary surgeons and students of veterinary medicine and show increased loads compared to other courses.

Major stresses or psychological resources during the working life of veterinary surgeons are primarily time, the freedom to make decisions and personal resilience as well as individual recovery times within the life areas of work, friends, family and the individual himself [4]. Long working hours, the work/life balance and private life, professional support (for example supervision, (psychological) advice from colleagues) and the expectations of the owners of patients are closely related to psychological well-being [10], [20]. This situation during the working day is reflected by investigations involving students who were found to suffer higher perceived stress, time pressure and depression [21], [22]. Various study-related and personal stressors were investigated, with most of them - such as for example a high work load, strict or unclear requirements, frequent exams, financial worries and relationship problems - not representing specific challenges that would occur only during the period of veterinary studies [6], [15], [18], [19], [21], [22], [23], [24]. 

Veterinary studies are regulated by the Veterinary Certification Act in Germany [http://www.gesetze-im-internet.de/tappv/BJNR182700006.html]. Following a statutory study period of 11 semesters, scientifically and clinically trained veterinary surgeons are supposed to be capable of working in their chosen profession independently and at their own responsibility. The practical part of their education covers 1,170 hours, whereby up to 850 hours are spent on a curative work placement. The aim of this practical training is, amongst other things, the acquisition of knowledge and clinical competencies. The European Association of Establishments for Veterinary Education (EAEVE), a European association whose task is the support and development of veterinary education within the European Union as well as the evaluation of European veterinary medicine education institutes, maintains a catalogue of competencies that should be acquired by the end of the course [http://www.eaeve.org/fileadmin/downloads/sop/SOP_Annex4to8_Hanover09.pdf]. The so-called “Day One Skills”, for example, are mentioned here, i.e. clinical capabilities and professional competencies such as for example medications and application types for various animal species. An investigation into the factual and social competence of young assistants in Germany found that a great number of clinical competencies of practising veterinarians should be classed as unsatisfactory [25], [26], [27]. Comparable results were obtained from investigations in the Netherlands and England [28], [29].

Data regarding the connection between the acquisition of competence and excessive stress right down to stress and complaints by students during their curative work placement is not currently available. It is the aim of this study to find out in which areas and to what extent competencies are acquired and psychological stress is suffered by students during the work placement. The following subject areas will be checked or questions dealt with as part of the same:

- Are students subject to excessive stress during their curative work placement?
- How is the relationship between the subjective acquisition of competence and excessive stress measured at the end of the work placement?
- What predictors (for example satisfaction, motivation etc.) reduce or increase a subjective acquisition of competence during the curative work placement?

## Material and methods

### Procedure and study design

Veterinary students were asked about work placement related resources and complaints as well as their connection with general conditions (semester attendance, institution of the work placement, period or working hours of the work placement) with the aid of an on-line questionnaire. Resources mentioned in this connection were inner strengths that would help a person to manage his workload in a way that enables the achievement of positively evaluated goals and an effective implementation. These can be character trails of the relevant person (capabilities, beliefs, talents etc.), but can also be external characteristics from the social (a harmonic relationship, support from colleagues etc.) and/or the material (money, living conditions etc.) environment of the person. Questions regarding the acquisition of practical competencies or questions regarding perceived excessive stress during the curative work placement were also looked at. The questionnaire was completed in an on-line format for every candidate in line with comparable criteria (presentation of the questions, fixed sequence of questions, information about the study itself etc.). A dedicated web-based survey tool was developed and programmed specifically for this study. The sample was recruited during a period from September to December 2014 (four months). The study as such was advertised during events at the participating universities. Participation in the questionnaire by the participants was voluntary and anonymous, and completion of the questions could be discontinued at any time. If they did answer all the questions the participants were given direct feedback concerning their results in the questionnaire.

#### Characteristics of the sample

The sample included n=142 participants, of these n=127 women and n=15 men. Only complete data sets were included in the data pool. The majority (n=101) of participants were single, n=33 lived in a long-term relationship, n=7 were married, n=1 divorced. 2% of participants were living with their children. The average age of the total sample was 25.3 years (SD=3.7). Students from all five veterinary universities or colleges of the following locations participated in the study: Hanover 59.9% (n=85), Leipzig 12.7% (n=18), Berlin 12.0% (n=17), Munich 12.0% (n=17), Gießen 3.5% (n=5). A division into the relevant study years or semesters at the time of the investigation resulted, for the 3^rd^ course year (up to the 6^th^ semester), in 14.1% (n=20), the 4^th^ course year (7^th^/8^th^ semester) in 21.1% (n=30), the 5th course year (9^th^/10^th^ semester) in 24.7% (n=35), the 6^th^ course year (11^th^/12^th^ semester) and more in 40.1% (n=57). The distribution of men and women as well as the age groups and the extent of resources and complaints etc. was comparable for the five educational institutes. The following results were therefore related to the total group of all students and were not broken down for individual universities.

#### Description of variables and instruments used 

**Figure 1:**
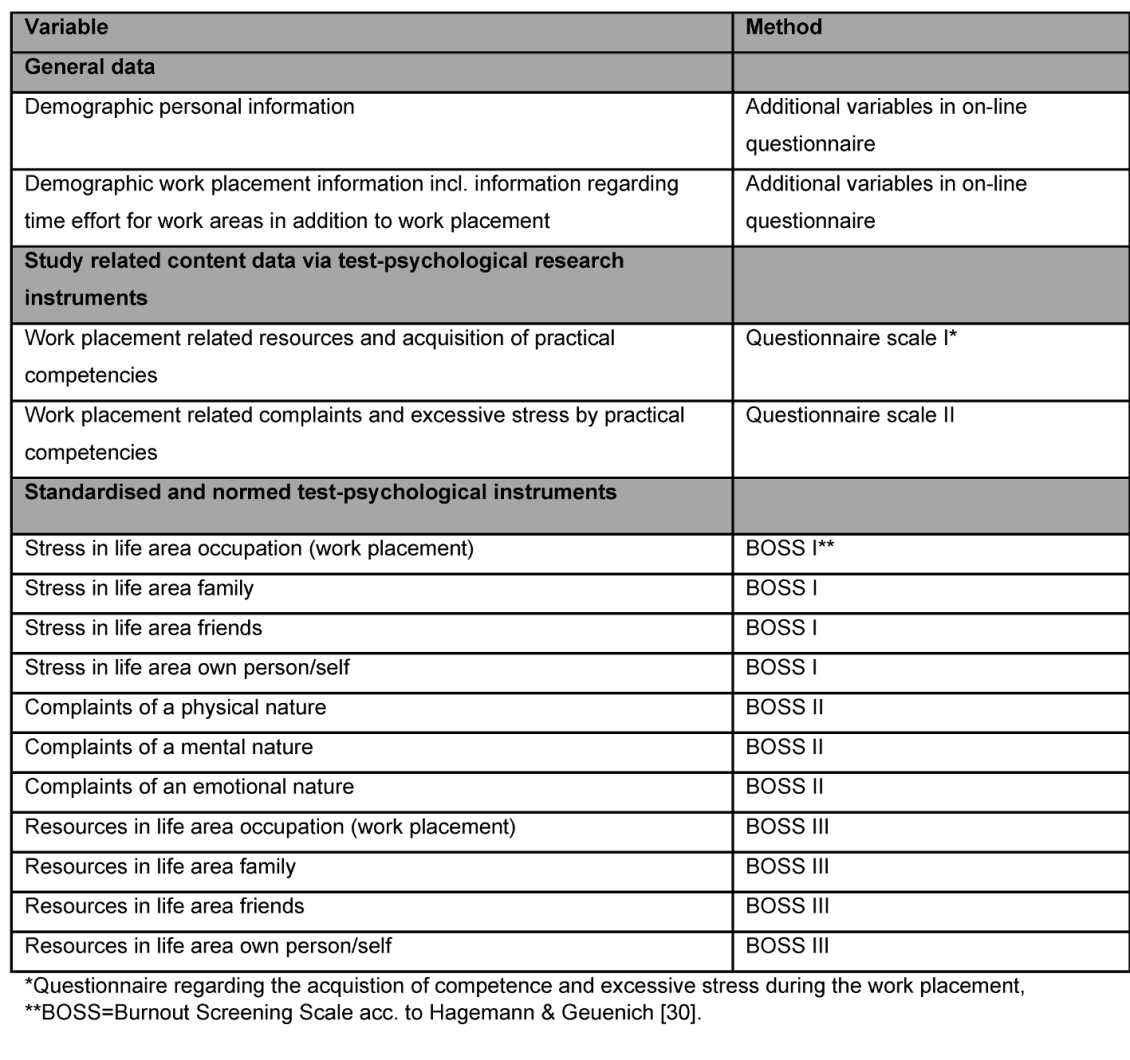
Illustration of variables and diagnostic methods included in the investigation.

The 137 characteristics recorded in the questionnaire firstly include demographic data and secondly scales that were developed as a research instrument and include work placement related resources and complaints. Burnout Screening Scales were also applied [30], [31]. BOS scales are standardised and normed, have been validated and checked to reflect suitability, resources, stress and psychosomatic complaints with clinical relevance, and were checked with the aid of a German language standardisation sample. The values and parameters included in the investigation as well as the above mentioned instruments with which the same were determined are listed in Figure 1 (Fig. 1) below and are outlined hereafter. 

**Figure 2:**
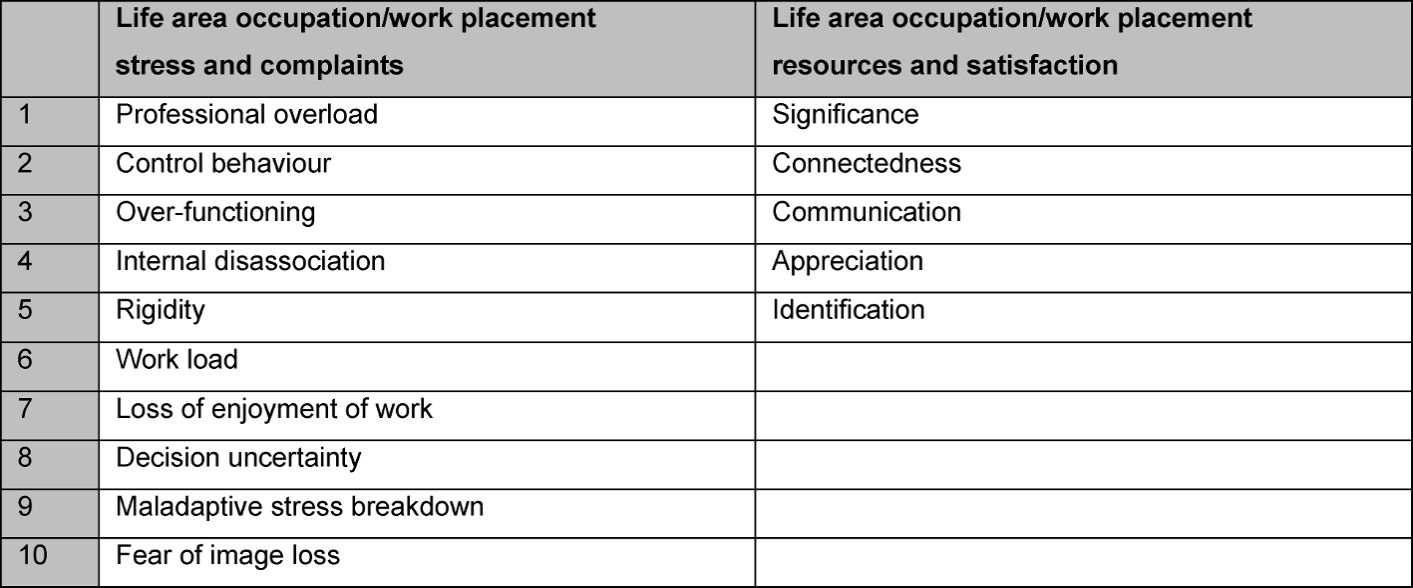
Summary of scale constructs of the scale work placement (adapted to scale occupation in BOSS I or BOSS III)

**Figure 3:**
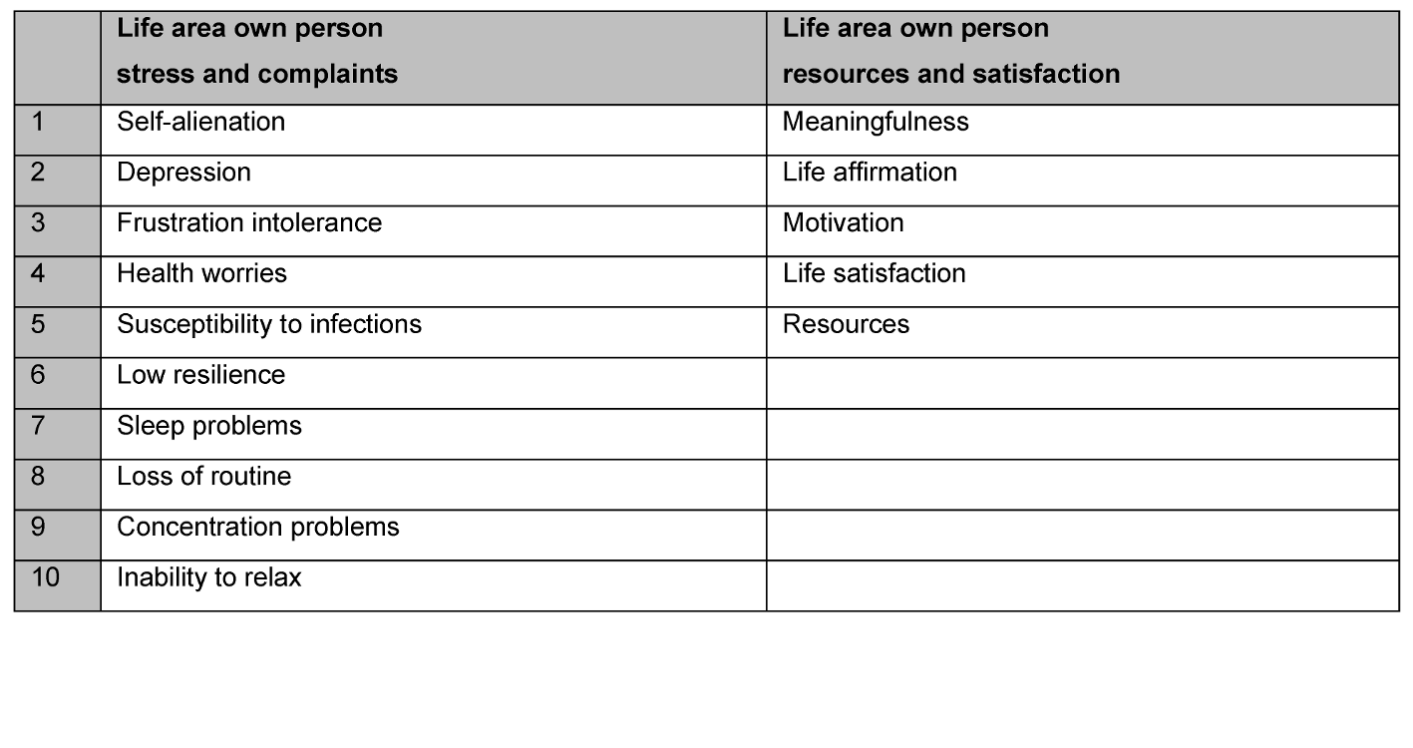
Summary of scale constructs of scale own person/self (BOSS I or BOSS III)

**Figure 4:**
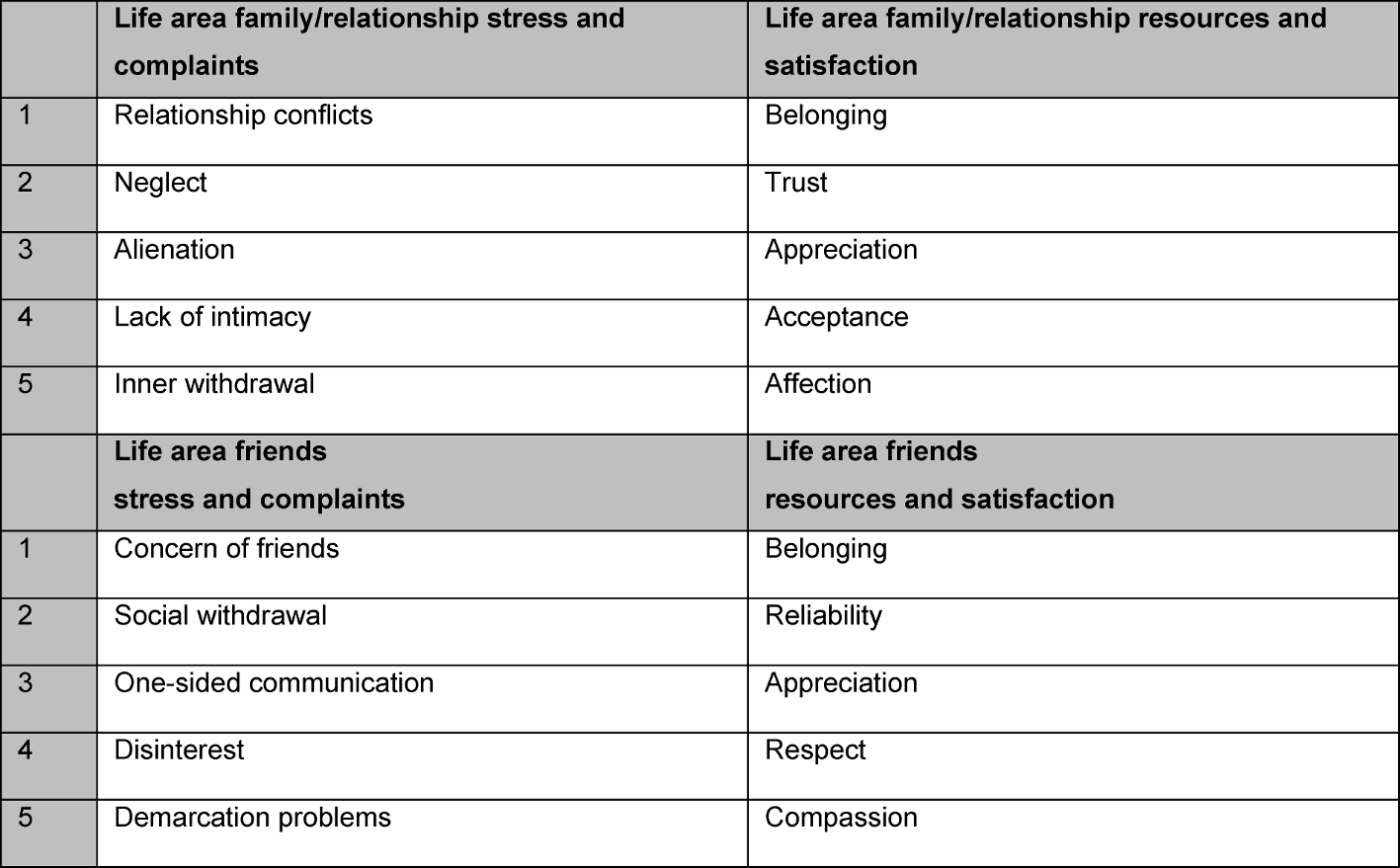
Summary of scale constructs for the scale family/relationship and friends (BOSS I or BOSS III)

**Figure 5:**
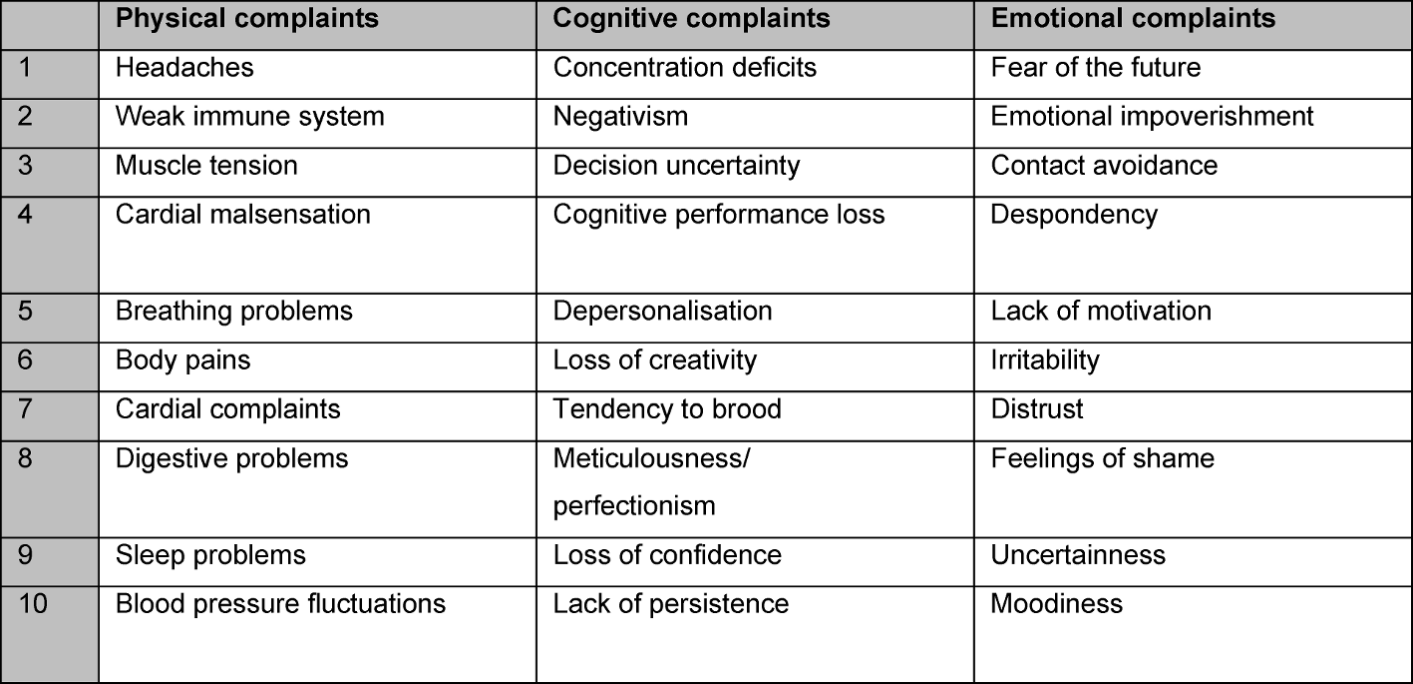
Summary of scale constructs for the scales physical, cognitive and emotional complaints (BOSS II)

Burnout Screening Scales (BOSS): burnout screening scales consist of the separately used questionnaires BOSS I, BOSS II and BOSS III. All three are self-assessment methods. The questionnaires BOSS I and II serve for recording current psychological (cognitive and emotional), physical and psychosocial complaints. BOSS III serves for recording resources. The scale constructs of the relevant BOS scales are shown in Figure 2 (Fig. 2), Figure 3 (Fig. 3), Figure 4 (Fig. 4) and Figure 5 (Fig. 5). As the scales relating to professions in BOSS I and III are not adjusted for the situation of a study course small terminology changes were applied here, for example: “occupation” was replaced with “work placement”, “colleagues” with “fellow students” etc. From a contents point of view, the constructs of BOSS I and III were not changed, so that we can assume that the standardisation values will still provide us with reliable evaluation parameters. 

Questionnaire BOSS II includes a total of 30 referral questions regarding clinical symptoms based on three scales of ten items each. It differentiates between levels of physical complaints and symptoms from the cognitive or emotional area.

#### Questionnaire for the acquisition of competence and excessive stress during the work placement

The questionnaire for the acquisition of competence and excessive stress during the work placement was developed as part of a joint project by Marc Dilly and Katja Geuenich in preparation for this study. It consists of two scales with a total of 44 items to be evaluated on a four-stage scale from 1-4 (see Attachment ).

**Figure 6:**
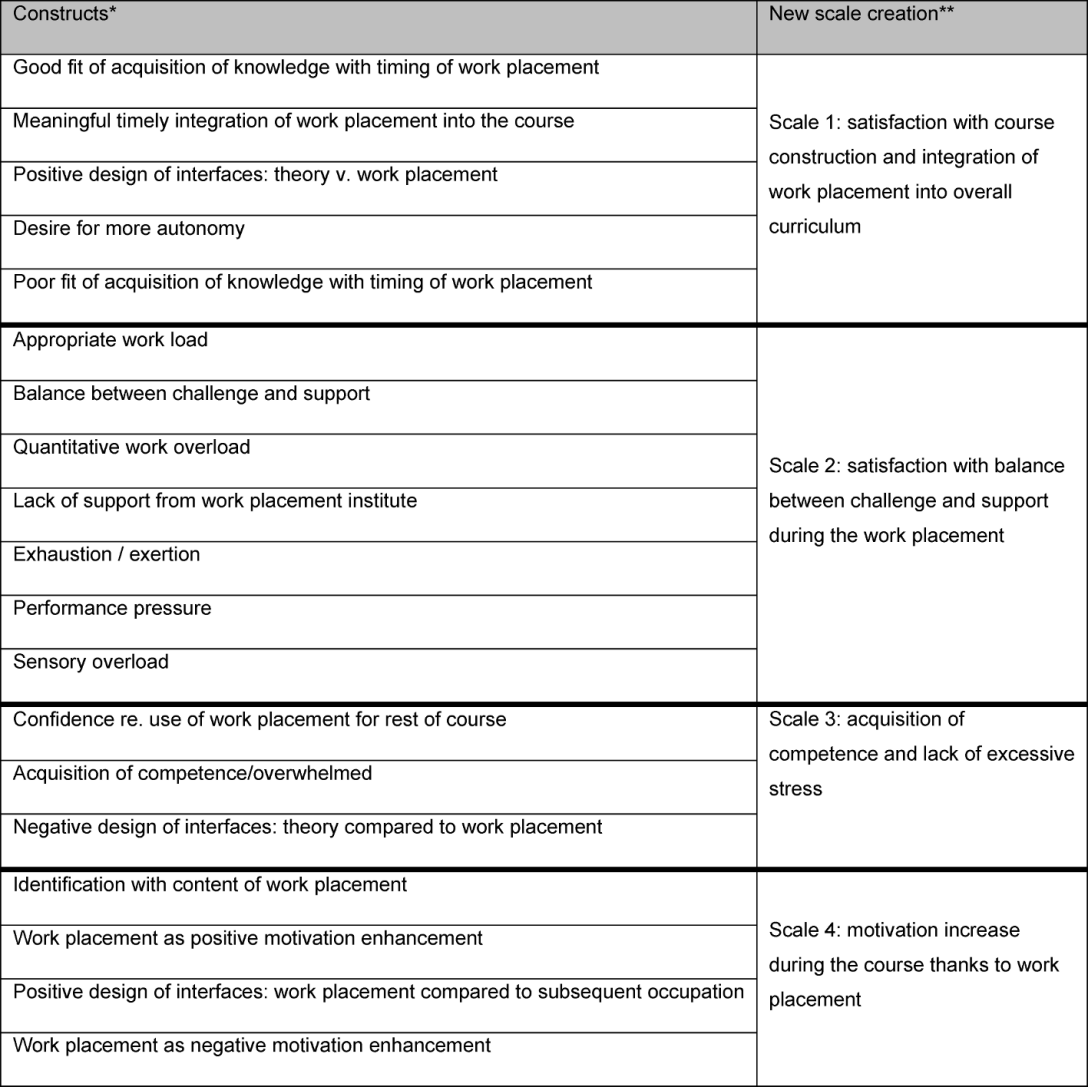
Summary of constructs and scale constructions of questionnaire compared with competencies and excessive stress during the work placement*. Constructs are not identical to the two factor: resources or excessive stress **. New scale construction implies a cross-factor content grouping of items.

The development of the scales was based on a number of working steps that build on each other. During a first working step an item pool of representative and characterising work placement related resources and obstacles comprising 30 items each was created on the basis of European requirements regarding practical competencies [http://www.eaeve.org/fileadmin/downloads/sop/SOP_Annex4to8_Hanover09.pdf] and expert assessments [28], [29]. During the next step a first reduction, oriented on terminology and formal requirements, was applied to the item total, whilst a third working section involved the submission of these selected questionnaire items to non-experts for evaluation in order to reduce the existing items once more time or reformulate them if necessary. The final version of the scales includes a set of 2 x 22 items. The internal consistency parameters of both scales are good. The following was calculated with the aid of the current sample of 142 students: the scale “resources and confidence during the work placement” includes all 22 items for consistency of the scale. The scale-related median alpha lies at α=0.90, within a satisfactory range. The scale “excessive stress and obstacles during the work placement” is similar, as the scale-related median alpha here lies even higher, at a value of α=0.92. Resources and confidence are here defined as mainly enduring talents, capabilities and the assessment of the acquisition of competences and practical capabilities inherent in the person during the curative work placement (see Figure 6 (Fig. 6)). The scale “excessive stress and obstacles during the work placement” defines excessive stress as an internal professional overload and workloads that can be more or less influenced by the person, whilst obstacles should be understood as external constructs that cannot be influenced by the person (see Figure 6 (Fig. 6)).

From the total of 20 (or incl. sub-scales=44 items) items, four new scales were created. The scale creation followed the contents logic or statement of the items and no statistical analysis. All four scales include items from the first part (resources and confidence during the work placement) of the questionnaire as well as the second part (excessive stress and obstacles during the work placement). The polarities of the items were adjusted to each other, so that high values in the new scale correspond to a high prevalence of the new characteristic. Figure 6 (Fig. 6) should be used for a comparison of individual items and the item numbers and construct names mentioned below.

#### Evaluation methods and statistics

In order to check the hypotheses and the results selected here from the calculation analysis the following evaluation methods were used, supported by the computer program SPSS (Statistical Package for Social Sciences): the detection of connections between work placement related resources and confidence as well as excessive stress and obstacles, and also between psychosomatic complaints and stress and resources, was primarily carried out with the aid of correlation, regression and factor analytical methods^1^. The scales determined or a priori ascertained in this way were then evaluated with regard to their internal consistency with the help of reliability analysis. Factor analysis was once again used for checking the internal structure of items of the newly developed questionnaire regarding the acquistion of competence and excessive stress during the work placement. Group differences and calculations regarding location-specific differences originate from T tests (incl. Levene test for variance identity). 

The requirements for use of computer applications were not consistently fulfilled, so that the requirement regarding proof of a normal data distribution, for example, which would not be expected with a self-selective questionnaire regarding the subject of stress and excessive stress during a study course, was indeed not present for most of the scales. The results should therefore be interpreted with a certain degree of caution, whereby the representation of data and the results calculated with their aid can be assumed thanks to the sample size of n=142.

The weighting of the Kaiser-Meyer-Olkin (KMO) criterion, which is not expected to be reached with the factor analysis of the questionnaire regarding the acquistion of competence and excessive stress during the work placement, could be omitted in favour of the internal consistency analysis. The consistency analysis shows whether items of a scale provide a substantial contribution or not. The KMO criterion records independence from variables. As psychological variables such as resources and excessive stress always assume the possible existence of common underlying constructs, for example exam anxiety, self-efficacy etc., which were however not checked here, a determination of partial correlations according to the KMO criterion was omitted. This also happened because an intercorrelation between the two scales or factors of the questionnaire regarding the acquistion of competence and excessive stress during the work placement, i.e. between resources and excessive stress, was expected. The scales created thus satisfy the criteria of internal consistency, given the current state of development of the questionnaire, but mostly complies with apparent content validated rather than statistical arguments. 

#### Data Protection Act

Participation in the study was anonymous and voluntary. All data recorded with the aid of the questionnaire was processed and evaluated in accordance with the Federal Data Protection Act. The method description was approved by the Data Protection Officer of the Foundation of the University of Veterinary Medicine of Hanover.

## Results

### Hours worked per week

**Figure 7:**
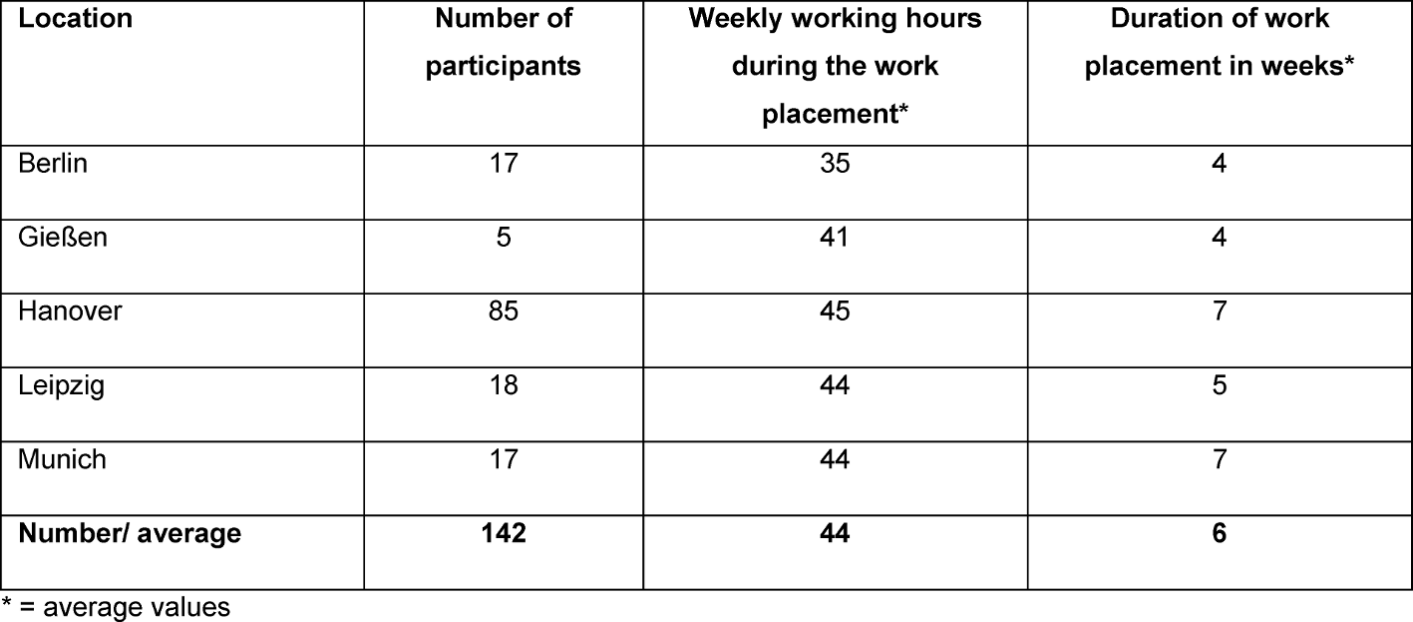
Information regarding participants with regard to hours worked per week and general conditions of work placement depending on the location.

The evaluation of information regarding hours worked per week and the total period of the evaluated part of the curative work placement at the relevant clinic or practice resulted in an average value of 44 working hours per week and an average total period of 6 weeks (see Figure 7 (Fig. 7)). The following details were provided by the participants, which are here averaged and related to the study: 35 hours or 4 weeks in Berlin, 41 hours and 4 weeks in Gießen, 45 hours and 7 weeks in Hanover, 44 hours and 5 weeks in Leipzig and 44 hours and 7 weeks in Munich.

#### Burnout Screening Scales (BOSS)

**Figure 8:**
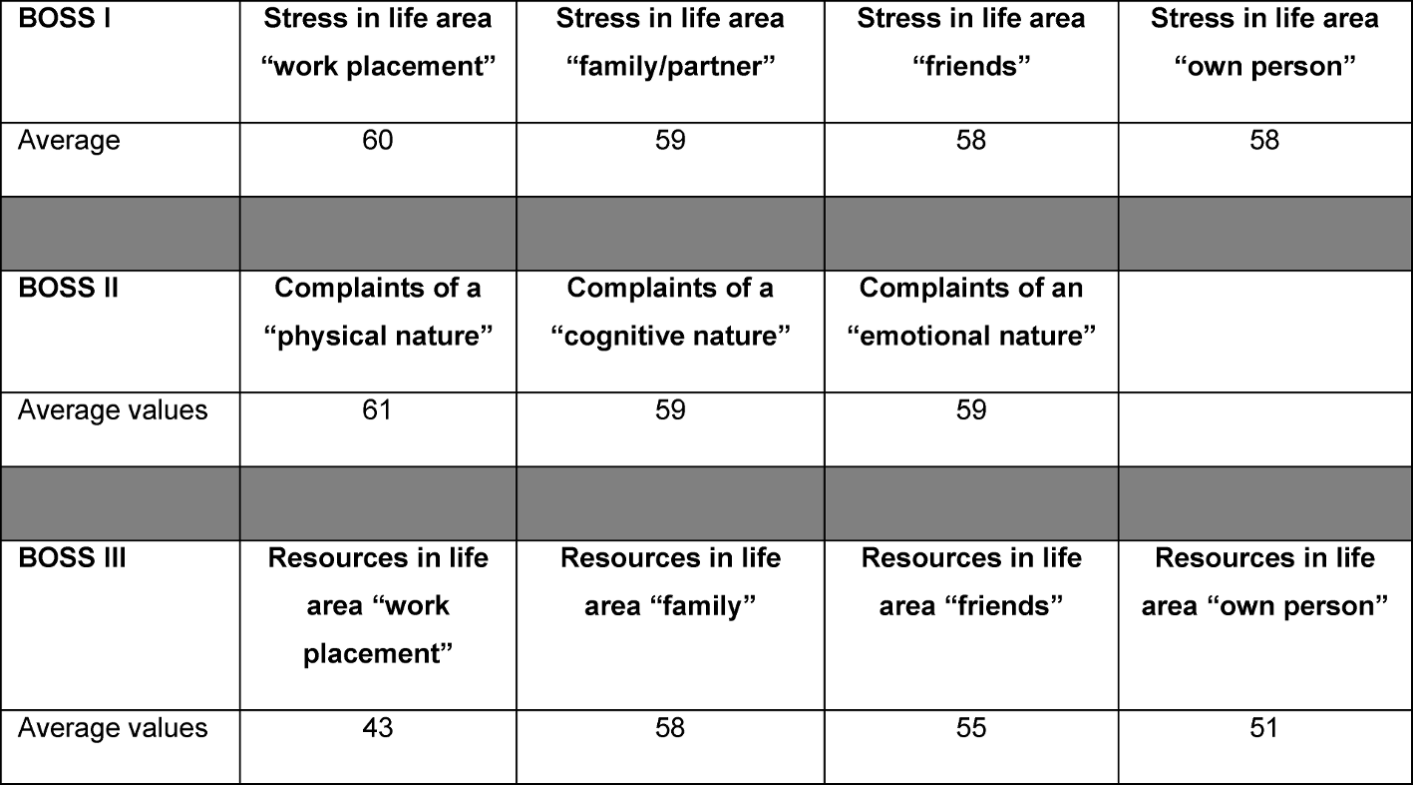
Summary of results (T values) of the Burnout Screening Scales (BOSS) I III of the students (n=142) during curative work placement; T values 20-40 are considered low, T values 41-59 are considered normal/inconspicuous, T values 60-64 are considered slightly raised, T values 64-69 are considered clearly raised, T values 70-75 are considered substantially raised, T values of 75-80 are considered very substantially raised.

The evaluation of BOS scales resulted in the following average values for all participants (see Figure 8 (Fig. 8)): BOSS I, in the sense of stress in the various areas of life, resulted in average values for the following areas: “work placement” 60 (min. 32. max. 80), “family/partner” 59 (min. 37, max. 80), “friends” 58 (min. 37, max. 77) and the area “own person” showed a median value of 58 (min. 35, max. 80). More than half of the participants (n=78) described a significantly increased load that deviated from the norm during their work placement. Complaints recorded for BOS II scales included average values for the following levels: “body” 61 (min. 38, max. 80), “cognition” 59 (min. 37, max. 80) and “emotion” 59 (min. 35, max. 80). Almost half of all participants (n=67) claimed a significantly increased psychosomatic load that differs from the norm. Results with regard to resources for BOSS III showed average values in the life areas “work placement” 43 (min. 28, max. 80), “family/partner” 58 (min. 25, max. 80), “friends” 5 (min. 31, max. 80) and that of the “own person” 51 (min. 27, max. 80). There was no significant difference in the BOSS results of the various locations.

**Figure 9:**
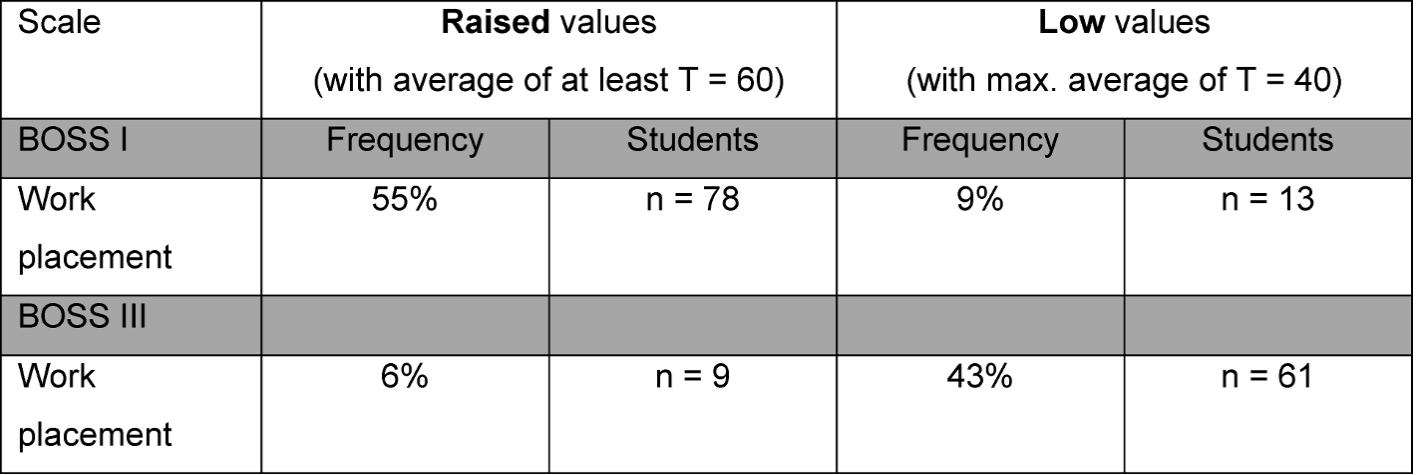
Summary of frequency and number of participating students (n=142) with raised or low values on Burnout Screening Scales (BOSS) I and III; T values 20-40 are considered low, T values 60-64 are considered slightly raised, T values 64-69 are considered clearly raised, T values 70-75 are considered substantially raised, T values of 75-80 are considered as very substantially raised.

Questionnaires BOSS I and BOSS III recorded stress and resources within the life area of the “work placement”. Figure 9 (Fig. 9) provides a summary of participants with significantly increased values (T values of at least 60) and lower values (T values of max. 40). 55% (n=78) of students questioned displayed a T value of at least 60 related to the curative work placement, which can be classed as an increased load. Increased resources of a T value of at least 60 were recorded by 6% of the participants (n=9). Lower T values of at least 40 were found for the area of stress (BOSS I) for 9% (n=13) or in 43% (n=61) of participants for the area “resources”.

#### Questionnaires regarding the acquisition of competence and excessive stress during the work placement

**Figure 10:**
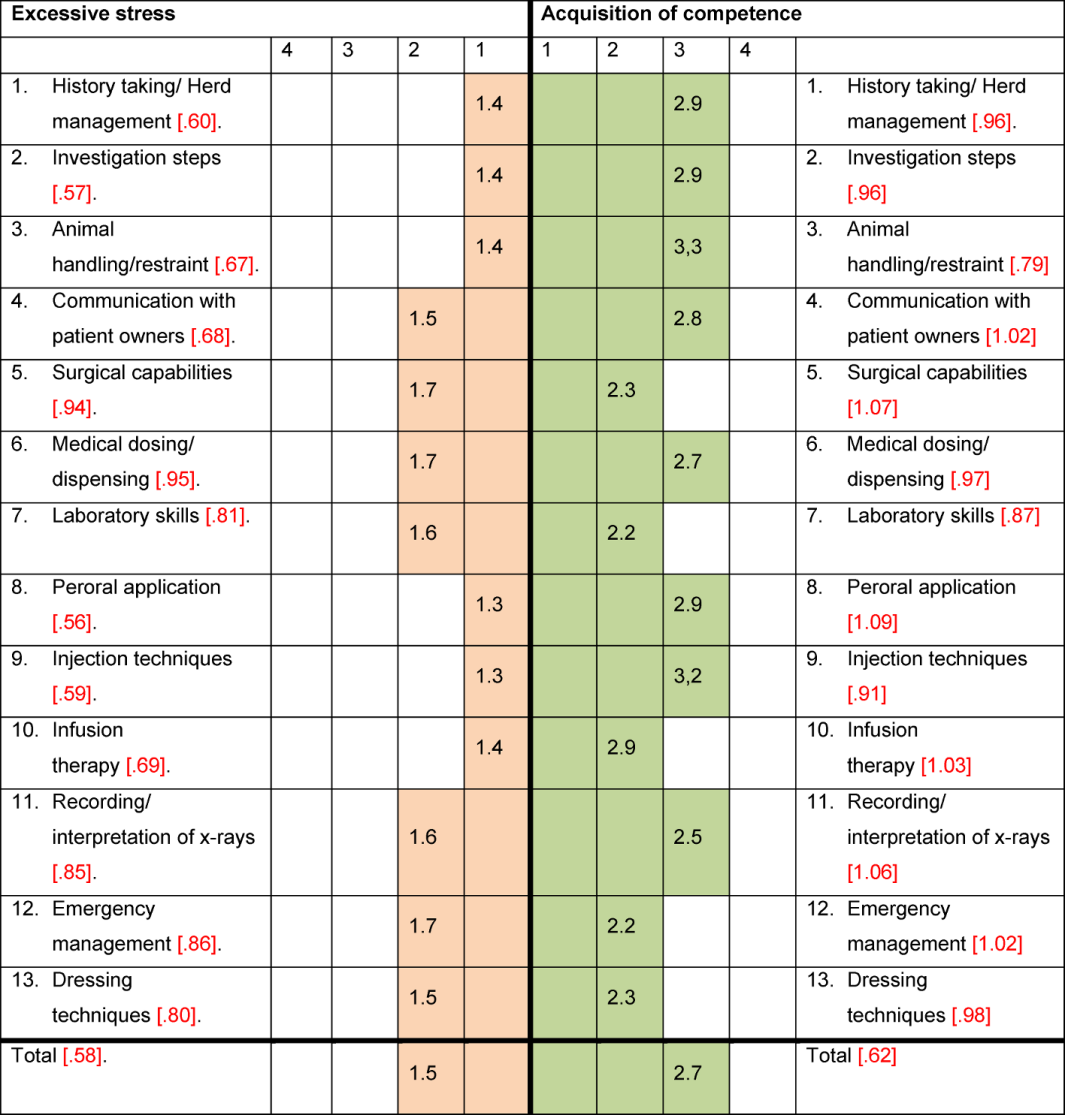
Average values compared with excessive stress in the practical capabilities area and acquisition of competence through practical capabilities of students (n=142) during curative work placement; value range of 1 to 4 with four answer alternatives 1 = is not true, 2 = is somewhat true, 3 = is mostly true and 4 = is definitely true; standard deviations in square/red brackets

The results of the questionnaire regarding the acquisition of competence and excessive stress during the work placement were evaluated on the basis of a four-stage scale (1=is not true, 2=is somewhat true, 3=is mostly true, 4=is definitely true). The following average values were determined for the scale “resources and confidence during the work placement”: fit between acquisition of knowledge during the course and during the work placement 3.4 (SD+/-0.79), suitable workload 3.0 (SD+/-0.91), meaningful timely integration of work placement into the course 2.9 (SD+/-0.86), balance between challenge and support 3.,1 (SD+/-0.82), use of work placement for the rest of the course 3.0 (SD+/-0.92), identification with content of work placement 3.2 (SD+/-0.90), work placement as a positive motivation enhancement 2.8 (SD+/-0.99), design of interface theory/work placement 2.1 (SD+/-0.82) and design of interface work placement/occupation 3.2 (SD+/-0.99). The ten items – whereby the 13 individual practical capabilities were assessed as one item, namely “practical competence”, here – significantly correlate with each other at 73% (r_min_=-.007, r_max_=.831. r_median_=.386). The results of the scale “excessive stress and obstacles during the work placement” are for the following constructs: fit between acquisition of knowledge during the course and during the work placement 2.3 (SD+/-1.12), quantitative professional overload 2.0 (SD+/-0.93), lack of support from work placement institute 1.7 (SD+/-0.96), exhaustion/exertion 2.1 (SD+/-0.98), performance pressure 1.8 (SD+/-0.89), work placement as negative motivation enhancement 1.6 (SD+/-0.90), desire for more autonomy 2.1 (SD+/-1.08), sensory overload 2.2 (SD+/-1.00) and design of interface between theory/work placement 1.9 (SD+/-0.93). At 84% these items intercorrelate significantly with r_min_=.062. r_max_=.660, r_median_=.345. The 13 capability-related items were also evaluated as one item here, namely as “excessive stress in practice”. Individual practical capabilities of scales “resources and confidence during the work placement” or “excessive stress and obstacles during the work placement” have been illustrated in a comparative way with regard to subjective excessive stress and acquisition of competencies during the curative work placement (see Figure 10 (Fig. 10)). These significantly correlate for the subjective acquisition of competence with 92% (r_min_=.114, r_max_=.729, r_median_=.345.) as well as for stress and obstacles with 100% (r_min_=.436, r_max_=.770, r_median_=.577). If we differentiate between the relationships of acquisition of competence with excessive stress during the work placement, all of these differences are positive in favour of the acquisition of competencies. The averaged values of the total sample as well as individual difference values of individual competencies exceed a subjective feeling of excessive demand. The three highest values for a competence increase were averaged for the following areas: handling of animal handling/restraint, peroral application and injection techniques. The lowest values were recorded for the areas of emergency management, surgical capabilities and laboratory skills.

The factor analysis of the questionnaire regarding the acquistion of competence and excessive stress during the work placement did not replicate (by means of the own value criterion) the two-dimensional form of the questionnaire with a separation into resources and excessive stress. The solution according to the own value criterion was a four-factor solution, which accounted for a total of 64% of the variance and resulted in a structure, the content of which covers the following four areas:

1. positive learning experience & value of work placement for the course (inherent value=6.5; resolved variance=36%),
2. stress and performance pressure (inherent value=2.3; resolved variance=13%),
3. interface work placement and theory course contents characteristics (inherent value=1.4; resolved variance=8%) and
4. interface work placement and theory course: structural characteristics (inherent value=1.2; resolved variance=6.9%).

As the previously defined two-factor solution was not confirmed, new scale constructs (similar to those of the four-factor solution with regard to content characteristics) were formed, with which explorative work was continued.

**Figure 11:**
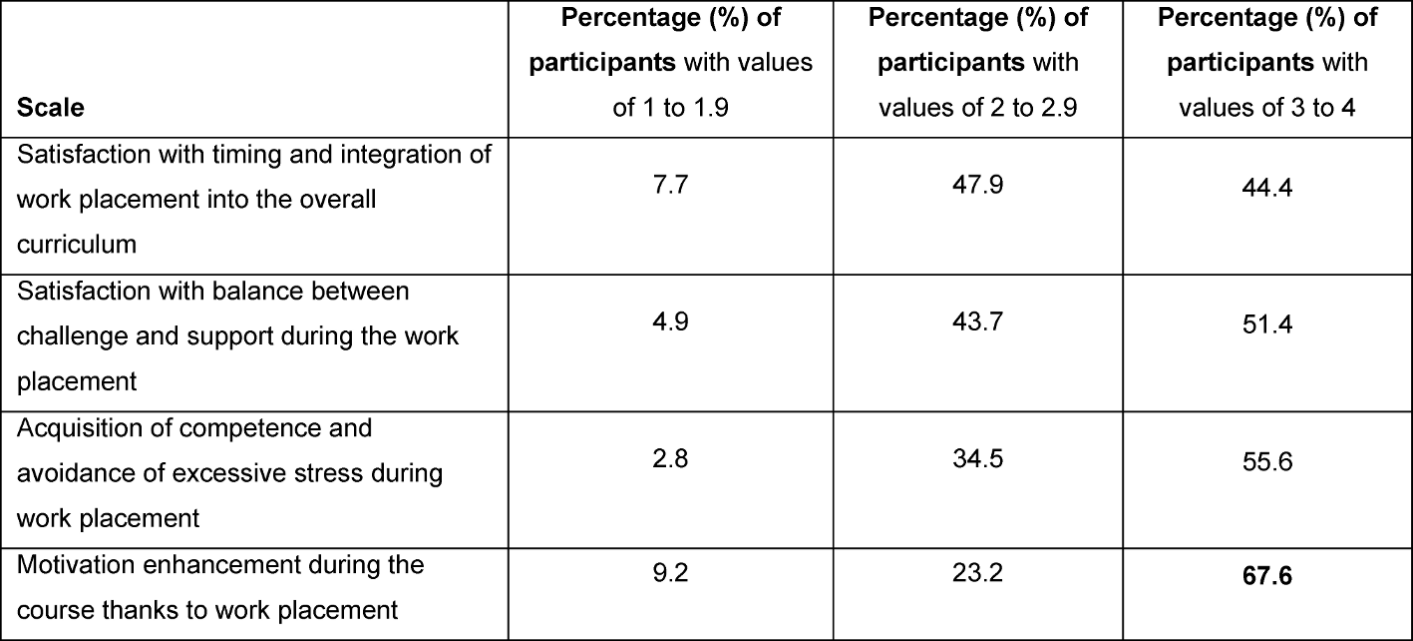
Scale constructs regarding satisfaction, acquisition of competence or excessive stress as well as motivation enhancement of curative work placement for students of veterinary medicine (n=142); value range of 1 = is not true, 2 = is somewhat true, 3 = is mostly true and 4 = is definitely true

44.4% of participants agree mostly or in every case (values from 3 in the answers scale of 1 to 4) with the statements of these newly formed scale constructs (see Figure 6 (Fig. 6)), namely “satisfaction with the timing and integration of the work placement within the overall curriculum”. “Satisfaction with the balance between challenge and support” was ascertained in 51% of participants at the same time. 55.6% of participants confirmed an acquisition of competence and avoidance of excessive stress mostly or in every case. Increased motivation during the course thanks to a curative work placement was confirmed by 67.6% of participants (see Figure 11 (Fig. 11)). The correlation between the scale constructs regarding satisfaction, acquisition of competence or excessive stress and of motivation enhancement is significant at a level of 0.01 (2-sided). 

Which characteristics can predict an acquisition of practical competencies? Which of them are – on the level of psychological and external conditions of the work placement, i.e. the characteristics of the questionnaire regarding the acquistion of competence and excessive stress during the work placement – predictors for a subjectively experienced growth of competence? It could be proven by means of regression analysis that three of the resource-related characteristics of the questionnaire can be considered as such predictors. These three characteristics are:

1. “Work placement as safe and helpful knowledge base for further studies”,
2. “Identification with the content of the work placement” and
3. “Experiencing a good balance between support and challenge during the work placement”.

The corrected R square is R^2^=.584. In line with the same approach, predictors for stressors representing excessive stress and learning obstacles were searched for on the other side. The following predictors are of importance here:

1. “Performance pressure and excessive stress (qualitative stress)” as well as
2. “High work density and scope (quantitative stress)”.

The corrected R square of the model here lies at R^2^=.297, which at least represents a relatively good predictability accuracy.

Participants (n=78) with increased loads during the work placement (BOSS I) differ significantly (p<.001) in their characteristics of satisfaction and acquisition of competences during the work placement from those participants who suffer less stress. Participants reporting significantly increased psychosomatic loads that vary from the norm (BOSS II) (n=67) also differ (p≤.002) in their characteristics of satisfaction and acquisition of competences during the work placement from those participants under a lesser load.

## Discussion

Empirical data regarding the extent of loads and resources, a possible overloading or signs of burnout in students of veterinary medicine during curative work placement, was recorded for this study. 

The following questions were investigated:

- Does overloading or do signs of burnout exist in students during curative work placement?
- What is the relationship between subjective acquisition of competence and excessive stress at the end of the work placement?
- Which predictors (for example satisfaction, motivation etc.) reduce or increase the acquisition of competence during a curative work placement?

It could be shown that more than half of the participants experienced increased loads during the work placement (BOSS I). This means that the loads described are higher for veterinary surgeons and human medicine practitioners than those with a normal working life [2], [4], [32]. Complaints and stress during the curative work placement accompanying the course go hand in hand with increased psychosomatic complaints in approximately half of the students (BOSS II). It thus transpired that complaints on a physical, emotional and cognition level mutually affect each other to an increased extent. Due to a suspected self-selection effect in the sample (voluntary participation) these values should be considered critical and it becomes clear at the same time that many students master the acquisition of practical competencies with motivation, but also with some effort (increasingly experienced in the form of stress and excessive stress).

One factor affecting the generation of these high values could be the fact that students already report significantly higher stress values or subjectively perceived stress compared to that of the normal population during their course, and cannot reduce this during the work placement or the work placement forms a part of this load situation [13], [16], [21], [33]. In order to prevent possible consequences such as for example substance abuse right up to suicide [5], [6], [16], [20], [34], [35], [36], measures and possibilities for active stress reduction, which should already have been acquired during the course, will be helpful for counteracting future stress during the work placement and subsequent occupation.

A curative work placement presents students with realistic situations in a professional context of the practising veterinary surgeon for the first time. It could therefore be assumed that students experience few situations during their course in which they would have to explain and be responsible for their own independent practical actions. Depending on their work placement position this can be subject to huge change – one is very much challenged here, many students experience for the first time in their life here what it means to carry out occupational tasks. If one also considers the curative work placement as a practice oriented and very realistic situation that is similar to the occupational working day of the practising veterinary surgeon it should be assumed that most students feel challenged, and some will feel overwhelmed by the acquisition and implementation of practical capabilities. It could also be assumed that a negative connection with the subjective learning success and the acquisition of competence exists in view of this challenge to the point of being overwhelmed. It has also been found that the ratio of subjective acquisition of competence to excessive stress can be perceived as positive for the acquisition of competence. The higher value of a subjective competence development compared to the lower values of perceived excessive demands thus go hand in hand for students during the curative work placement. At the same time one can imagine a negative correlation that works against the acquisition of competence if excessive stress is caused by a lack of, or by suppressive supervision by the monitoring veterinary surgeon. The preparation of guidelines and the upkeep of a diary or “log book” during the work placement as well as fixed discussion and reflection meetings during the work placement would represent one opportunity for optimising and regulating a subjective as well as a more objective acquisition of competence. 

In this way the work placement is balanced in a positive way despite the high stress level reported by many students. Data confirms that too much stress (measurable by means of explicit descriptions of stress and also in view of increased psychosomatic symptoms; BOSS I and II) is associated with a low acquisition of competencies and not only increases emotional stress, but also doubt as to whether the right occupation has been chosen, a fall in motivation and unhappiness with the course. It should therefore be considered here how it can be recognised early on that critical eustress limits have been exceeded and have been replaced with distress that represents a risk to health. We should consider mentors or even consultation for students during the work placement.

Students are meant to expand their competence during the work placement and as part of their course. This includes not only knowledge and practical experience, but also the handling and solving of clinical cases during the work placement. The increase in practical competence subjectively perceived by students rose as the work placement progressed. At the same time increasing work loads and longer working hours also resulted in increased stress loads. Weekly working times averaged 44 hours as a quite realistic range, compared with weekly working hours of 45-55 hours for practising veterinary surgeons [9]. The length of the working week transpired to be one of main reasons for stress for the practising veterinary surgeon suring several studies [10], [11],[20]. It was not possible to replicate this in the same way for this study. 

From the point of view of practising vets, veterinary education courses should include more practice related work [25], [27] and students of veterinary medicine also suggest more practical experience during the course as an option for reducing stress [13], whereby it can be assumed in the latter case that students are not thinking of the demands of the learning process here, but of an improved learning basis following the successful acquisition of practical competencies. Earlier studies confirmed similar tendencies, where practice related teaching courses resulted in increased motivation and eagerness to learn in students [37], [38], [39]. In addition, it was found that practice related changes to the curriculum can contribute towards a subjective stress reduction for students [40], [41], [42], [43], [44]. It remains unclear, however, to what extent the average period of six weeks can have a positive or negative influence on loads and resources. Studies have shown that a period of at least three weeks has a postive effect on the acquisition of practical capabilities within the surgical area [45], whilst long-term intervention implemented during the current curriculum have found to be promising with regard to the acquisition of clinical and practical skills [42], [46].

It can also be assumed that many students experience increased levels of expectation and that their expectations therefore rise, resulting in increased performance pressure. This is confirmed by the results of numerous earlier studies, in which performance pressure has often been mentioned as a trigger for stress by students of veterinary medicine [18], [19], [22]. We can further assume – and this has been confirmed by this study – that this performance pressure is associated with stress, whereby - as has already been mentioned - a direct and significant connection was barely noticed on the other hand with regard to the acquisition of competences. The extent to which competencies are acquired will depend on a high identification with the work placement and occupation in general as well as a good balance of challenge and support [13]. The work placement should be viewed as a kind of “protected” area of the students education here. It revolves around the opportunity of acquiring or extending competencies and gather practical clinical experience at the same time. Despite this the gathering of experience cannot be compared to learning. It would thus be expedient for the orientation of students to combine definite learning targets for individual work placementsand to intensify the cooperation between universities and work placement organisers in order to support practical learning that can also be evaluated with the aid of objective data. The “Guideline for learning targets during the work placement” of the Association of Practising Veterinarians (bpt) [http://www.tieraerzteverband.de/bpt/Studenten/ausbildungspraxis/09_10_Leitfaden_fuer_das_tieraerztliche_work placement.pdf] represent a good starting point for such an orientation and comparision of teaching and learning targets. A number of further measures such as the qualification measures of lecturers with regard to teaching, pastoral care and the advising of students as well as the introduction of clinical rotations or a “practical year” might contribute towards a good preparation for a work placement or the start of occupational work [47], [48], [49], [50]. Long-term studies would be helpful here to ascertain the effect of the measures described on occupational beginners. It can be assumed that a good integration of the work placement into the curriculum will result in less stress for students. The Veterinary Cerification Act (TAppV) stipulates the period of the various work placements during the practical part of the course as a total of 1,170 hours [http://www.gesetze-im-internet.de/tappv/BJNR182700006.html]. These must include the completion of at least two curative work placements including a total of at least 500 hours and a maximum of 850 hours as part of the veterinary education. The first work placement at a curative practice or clinic includes 150 hours, whilst a second one will take at least 350 to 700 hours, depending on the duration of an optional work placement. The timing of these curative work placements during individual course sections specified by the course curriculum would therefore appear crucial. Thanks to different course organisation and differing applications of the testing clause (TAppV, section 3) and the organisation of optional work placements by the various educational authorities, heterogeniety connected with the same is given for the various education authorities. In addition the work placement location or the organisational structure of the work placement location can have a more or less strong influence. It can thus be assumed that the (university) clinics of the education authorities would provide good organisational structures for students, whilst private clinics, centres, individual practices or relationships with limited personnel structures would have and provide less opportunity. Individual care and education as part of a good working relationship in smaller practices should be postulated for smaller practices. 

One limitation of the study is the fact that the on-line questionnaire stipulates a selection of competencies or areas of competence. Selections by means of the EAEVE stipulations and the established Day One Skills catalogues were stipulated at the same time [http://www.eaeve.org/fileadmin/downloads/sop/SOP_Annex4to8_Hanover09.pdf], [28], [39]. A study with a qualitatively oriented approach (for example by means of guideline interviews, evaluation of video recordings etc.) would be required for differentiating between results. Further limitations of the study are - apart from the instruments selected - the assumed self-selection effect on participants already mentioned above, and with it a distortion of the data distribution. An important reason for the decision to use BOS scales in place of the also well-established Maslach Burnout Inventory (MBI) [51], [52] was the lack of norming of a German language sample of the MBI. A German language comparison group exists for the BOS scales [30]. There are also several German studies that involved the recording of stressors and resources in veterinary and human medicine that use the BOS scales. A direct comparison was therefore possible.

## Conclusion

A large number of students of veterinary medicine display increased loads up to increased psychosomatic complaints during their curative work placement, which are comparable with the loads described for veterinary surgeons during their everyday working life. The students questioned with regard to practical capabilities to be learned mentioned relatively little stress thanks to a high subjective acquisition of competences. The highest values in the sense of perceived excessive demands were described for the areas of emergency management, surgery and medication dispensation - whilst information relating to the acquisition of competence simultaneously exceeded those concerning excessive stress related to content. The highest values were recorded for the areas of animal handling/restraint and application and injection techniques. It can therefore be assumed for these latter areas that they are very well taught during the course or that sufficient opportunity exists during the work placement to acquire them. Overall the acquisition of practical competencies is considered as more important than a feeling of being overwhelmed by the students. The acquisition of practical competencies can thus be best predicted with the aid of the following characteristics: “work placement as a safe and helpful knowledge base for the rest of the course”, “identification with the work placement content” and “experiencing a good balance between support and challenge during the work placement”, whilst stress can be predicted with the aid of factors, performance pressure and perceived stress. Intervention on the part of students, but also from the point of view of the work placement institutes and the lecturers would be excellent starting points here.

In general, and by way of limitation, it can be said that a certain self-selection effect must be assumed due to the voluntary participation in the study, which would initially limit the statement value of the data. The results of this study, much like those of other investigations regarding stress and complaints, include some distortion of distribution caused by this effect. At the same time, we have however established some idea of resources during the curative work placement, possibilities for a balanced ratio of support and challenge. The inclusion of specific questions regarding the load situation of students offers evaluation and personal consultation possibilities for individual care purposes, with the aim of identifying possible complaints and be able to counteract the same. With regard to the acquisition of competence and the load structure of students during the work placement more support for the individual and fewer changes to the organisation of the work placement during the course are desirable and promising, as already described above.

## Notes

^1^ Correlation (double-sided, acc. to Pearson), factor analysis via the main component analysis with varimax rotation, linear regression analysis, reliability analysis by means of model α. 

## Acknowledgements

The authors would like to thank all participating students as well as the university professors/deans, in particular Prof. Dr. Dr. Stefan Arnhold and Prof. Dr. Christoph Mülling, for their support with this project. In particular, we would like to thank Dr. Christina Beitz-Radzio for her support in Munich and Stephan Birk for his help in Berlin. The project was sponsored by the Competency Centre for E-Learning, Didactics and Educational Research into Veterinary Medicine (KELDAT).

## Competing interests

The authors declare that they have no competing interests.

## Supplementary Material

Questionnaire for the acquisition of competence and excessive stress during the work placement
