# Supplementary material for: Stressed out or subjective acquisition of competence – how do veterinary students see their curative work placement?
Source: GMS J Med Educ. 2016 Feb 15;33(1):Doc9. doi: 10.3205/zma001008 (PMC4766928; doi:10.3205/zma001008)
Supplement: Questionnaire for the acquisition of competence and excessive stress during the work placement [file JME-33-9-s-001.pdf]

Questionnaire regarding the acquisition of competence and excessive stress during the work placement (FKÜP)

Demographic characteristics:

Age: ____________ Gender: ◻ female ◻ male

Status: ◻ single ◻ married ◻ divorced ◻ in a long-term relationship

I have children ◻ yes ◻ no

The children and I live in the same household ◻ yes ◻ no

On the next few pages you will find questions and statements regarding stress and resources encountered during the work placement recently completed by yourself.

Please answer these for the **last two to four weeks** with regard to how strongly the stresses or resources mentioned apply to you. Please use a scale of 1 to 4, i.e. use one of four alternative answer each time: 1 = is not true, 2 = is somewhat true, 3 = is mostly true and 4 = is definitely true/I strongly agree.

Please select the answer alternative that applies to you most for each statement and mark your answer clearly with a cross next to the relevant number or in the box intended for this purpose. Please do not place crosses between the answer alternatives. Please answer all the questions.

General conditions of the work placement

I am attending the ___________ semester

At which institute did you complete your last work placement?

____

In what area will you complete your next work placement?

____

How many weeks did your work placement last? _______ weeks

How many hours per week did your work placement consist of? _______ hours per week

How many additional hours did you spend per working week on average on

1. Learning and exam preparations _________ hours.
2. Completion of final thesis (seminar work, dissertation etc.) _________ hours.
3. Part-time job outside of my course/work placement _________ hours.

	1 = Is not true	2 = Is somewhat true	3 = Is mostly true	4 = Is definitely true
**8. I feel that the allocated work placement period and the timing of my last work placement during the course were appropriate and easy to manage.**	1	2	3	4
**9. I feel that the demands of the work placement allocated by the work placement organiser/period and the placement of the work placement within the plan of the overall course resulted in stress and excessive workloads.**	1	2	3	4
**10. I feel that the selection work placement times (placement of work placement within the plan of the overall course) was selected sensibly.**	1	2	3	4

1. Resources and stress during the work placement

5

**Resources and confidence during the work placement (acquisition of competence)**<br>***The following questions relate to the work placement recently completed by yourself:***	1 = Is not true	2 = Is somewhat true	3 = Is mostly true	4 = Is definitely true
**1. My work placement provided adequate challenges and support.**	1	2	3	4
**2. My work placement enabled be to prepare well and gave me skill qualifications for the rest of my course.**	1	2	3	4

**3.**	**My work placement helped me to improve my practical competence for the veterinary profession by means of the following capabilities:**				
	**a. Anamnesis recording/resource management**	1	2	3	4
	**b. Examination processes**	1	2	3	4
	**c. Handling of animals/compulsory measures**	1	2	3	4
	**d. Communication with patient owners**	1	2	3	4
	**e. Surgical capabilities**	1	2	3	4
	**f. Medical dosing/medical dispensation**	1	2	3	4
	**g. Laboratory skills**	1	2	3	4
	**h. Peroral application**	1	2	3	4
	**i. Injections**	1	2	3	4
	**j. Infusion therapy**	1	2	3	4
	**k. Recording/interpretation of x-rays**	1	2	3	4
	**l. Emergency management**	1	2	3	4
	**m. Dressing techniques**	1	2	3	4
**4.**	**I was able to identify well with the work placement and its content.**	1	2	3	4
**5.**	**Following the work placement, I am highly motivated for the next semester.**	1	2	3	4
**6.**	**I was well prepared for the requirements of the work placement thanks to the semester that preceded the work placement.**	1	2	3	4
**7.**	**The work placement has once again confirmed my choice of occupation as correct.**	1	2	3	4
**8.**	**I noticed during the work placement that I will be able to acquire the practical capabilities needed by a veterinary surgeon.**	1	2	3	4

**9.**	**I enjoyed the work placement.**	1	2	3	4
**10.**	**The possibility of working as a veterinary medicine practitioner has become more clearly defined and more realistic thanks to the work placement.**	1	2	3	4

**Excessive stress and obstacles during the work placement** *The following questions relate to the work placement recently completed by yourself:*	1 = Is not true	2 = Is somewhat true	3 = Is mostly true	4 = Is definitely true
**1. I feel poorly prepared for the next semester by the work placement.**	1	2	3	4
**2. The work placement was stressful for me due to excessive quantitative stress (working times, workloads etc.).**	1	2	3	4
**3. The requirements of the work placement were too high. This applies to the following areas:**				
**a. Anamnesis recording**	1	2	3	4
**b. Examination processes/resource management**	1	2	3	4
**c. Handling of animals/compulsory measures**	1	2	3	4
**d. Communication with patient owners**	1	2	3	4
**e. Surgical capabilities**	1	2	3	4
**f. Medical dosing/medical dispensation**	1	2	3	4
**g. Laboratory skills**	1	2	3	4
**h. Peroral application**	1	2	3	4
**i. Injections**	1	2	3	4
**j. Infusion therapy**	1	2	3	4
**k. Recording/interpretation of x-rays**	1	2	3	4
**l. Emergency management**	1	2	3	4
**m. Dressing techniques**	1	2	3	4

**Excessive stress and obstacles during the work placement**<br>***The following questions relate to the work placement recently completed by yourself:***	1 = Is not true	2 = Is somewhat true	3 = Is mostly true	4 = Is definitely true
**4. I am disappointed with the engagement of the work placement institute**	1	2	3	4
**5. The work load of the work placement was more of a challenge than I had anticipated.**	1	2	3	4
**6. I was subjected to great performance pressure during the work placement.**	1	2	3	4
**7. I am becoming increasingly less motivated and see the next work placement as more of a strain.**	1	2	3	4
**8. I would learn more if I could act autonomously and design my work placement on a more individual basis.**	1	2	3	4
**9. I had the impression that my head was too full up with all the information I had to process during the work placement.**	1	2	3	4
**10. The transition from the last semester to the work placement was like jumping into icy water.**	1	2	3	4
